# Understanding the Relationship Between the Neurologic Pupil Index and Constriction Velocity Values

**DOI:** 10.1038/s41598-018-25477-7

**Published:** 2018-05-03

**Authors:** Ifeoluwa Shoyombo, Venkatesh Aiyagari, Sonja E. Stutzman, Folefac Atem, Michelle Hill, Stephen A. Figueroa, Chad Miller, Amber Howard, DaiWai M. Olson

**Affiliations:** 1 School of Medicine, University of Texas Southwestern, Dallas, TX USA; 2 Department of Neurology & Neurotherapeutics, University of Texas Southwestern, Dallas, TX USA; 3 Department of Neurological Surgery, University of Texas Southwestern, Dallas, TX USA; 4 Department of Biostatistics, University of Texas Southwestern, Dallas, TX USA; 5 Neurocritical Care, Riverside Methodist Hospital, Columbus, OH USA

## Abstract

The pupillary light reflex (PLR) describes the response when light hits the retina and sends a signal (cranial nerve II) to the Edinger-Westphal Nucleus which via cranial nerve III results in pupillary constriction. The Neurological Pupil index^TM^ (NPi) and pupil constriction velocity (CV) are two distinct variables that can be observed and measured using a pupillometer. We examine NPi and CV in 27,462 pupil readings (1,617 subjects). NPi values <3.0 and a CV < 0.8 mm/sec were considered abnormal. Regression was used to clarify the effect of pupil size and repeated measures. An odds ratio of abnormal CV given normal NPi (and vice versa) was computed using the glimmixed (SAS) regression. Of 27,462 readings, 49.2% revealed bilaterally normal NPi wtih brisk CV, and 10.8% revealed bilaterally abnormal NPi and slow CV; 9.1% with unilaterally normal NPi and brisk CV where the opposite pupil had an abnormal NPi and slow CV. The remaining 30.9% revealed that one or both PLR had either a normal NPi with slow CV, or abnormal NPi with brisk CV. Brisk CV does not rule out an abnormal PLR; slow CV does not rule in abnormal PLR. Practitioners should consider these implications when interpreting pupillometry readings.

## Introduction

Handheld automated pupillometry is now widely available and commonly used in critical care settings^1–4^. Before this device became popular, assessment of the pupillary light reflex (PLR) in critical care was dependent on subjective assessment. Practitioners were taught to interpret the PLR as brisk, sluggish, or non-reactive^5^; relatively ambiguous terms that have been found to be unreliable^6^. The shortcomings of human derived subjective measures provided grounds for hand-held pupillometry. Automated pupillometer using the Neuroptics® NPi 200-pupillometer provides a precise measure of the constriction velocity (CV), latency, dilation velocity, size, percent change, and a Neurological Pupil index^TM^ (NPi). The NPi is a scaled value that is based on a model of normal pupilometer values^1^. As with any technological advance, a more thorough understanding of the measures obtained is required to make inferences regarding said measures. Recently, the authors of this manuscript observed patients who had briskly reactive pupils, but abnormal NPi readings. The purpose of this analysis is to examine the prevalence of brisk CV in cases with abnormal NPi in a neurocritical care population.

## Background

The eyes have always been a medical and an artistic infatuation^7,8^. From the age of enlightenment, through the renaissance, to this modern period, the eyes continue to be an object of fascination. The PLR pathway has been previously described with relevant diagrams^9^. In brief, the quantum energy in light elicits retinal nerve signals from the fovea, bipolar and amacrine cells, which relate visual information to the retinal ganglion cells^10,11^. The axons of these ganglion cells project to the nuclei of the oliviary pretectal nucleus, which connects to the Edinger-Westphal nucleus (EWN)^4,12^. The EWN is a preganglionic parasympathetic nucleus that projects efferent axons to the ipsilateral ciliary ganglion (IPSG)^13^. Post-ganglionic fibers carry signals from the IPSG to the constrictor muscles of the iris and cause pupillary constriction^13^. Hence, the eyes, which are truly an extension of the brain, provide information about the neuronal dynamics of both hemispheres of the brain. The PLR is therefore, an important and non-invasive element of neurological physical examinations^14^.

For centuries, pupillary reflexes have been investigated using subjective measures of pupil size, shape, and reactivity. Manual observation was the default method for assessing the PLR, especially in the setting of an acute neurological change^14,15^. Yet, manual evaluation of the pupillary reflex has been shown to have a very low reliability and validity^6,16^. Clark *et al*. indicate that manual pupillary examinations also contribute to prognostic findings that are limited to correlations between unresponsive pupils and negative outcomes^17^.

On the other hand, automatic pupilometers have been shown to have a high interdevice reliability^18^. The conventional variables associated with the output of the automatic pupillometer have also gained popularity in clinical applications^17^. These variables: maximum size, minimum size, CV, latency, and the NPi have been assessed and discussed across various studies^19^. The NPi formula is derived from a proprietary algorithm and reflects a comparison of the sum of all measured variables in the PLR in comparison to known normal observations^20^. An NPi of less than 3.0 is considered abnormal^19^.

The CV reflects the change in pupil size from baseline to smallest value over time and is determined in part by numerous benign environmental conditions such as ambient light and also by baseline pupil size^21–23^. A lack of research defining a true normal value for CV led us to dichotomize CV at 0.8 mm/s based on preclinical, historical evidence and clinical experience as roughly 2 standard deviations from mean values in healthy populations^21–23^. Therefore, the expectation that an NPi of less than 3.0 automatically corresponds to lower CV values (sluggish pupils) is not neccesarily a valid assumption. However, there is a gap in the literature examining this relationship.

## Methods

This analysis examines data from the Establishing Normative Data for Pupillometer Assessments in Neuroscience Intensive Care (END-PANIC) Registry. The design and methods for the END-PANIC registry have been previously described^1^. In brief, END-PANIC is a multicenter prospective registry of automated pupillometer readings and associated measures, which was implemented in order to collect adequate data that will form a normative database generating near population-values for all variables that are associated with PLR. The registry includes patients with diagnoses of acute ischemic stroke (AIS), intracerebral hemorrhage (ICH), subarachnoid hemorrhage (SAH), neoplasm (tumor), or other neurosurgical injury (NSU) requiring admission to a neurocritical care unit.

Pupillometer exams are standard of care in the participating neurocritical care units. Therefore, all patients admitted to neurocritical care were included if they had orders for the nurse to assess the PLR. The study was carried out in accordance with all state and federal regulations and was reviewed by the University of Texas Southwestern Institutional Review Board (#062015–005) as exempt from written consent (45 CFR 46). This analysis, includes 27,462 pupillometer readings from 1,617 de-identified individuals who met the criteria for inclusion of age older than 18 and were admitted for neurocritical care. Statistical analysis was performed using SAS v9.4^24^.

Data were sorted by a subject while retaining key variables: NPi, CV, and size, for both left eye and right eye independently. The variables associated with each subject were transformed into dichotomous groups of categorical variables, which categorized each output as either normal or abnormal. Clinically, constriction is historically graded as brisk, sluggish, fixed^25^. A value < 0.8 mm/s was used to dichotomize CV as sluggish based on data from the END-PANIC registry^1^ (2 standard deviations from the mean) and prior evidence in health populations, including preclinical data^23,26–28^.

To more fully examine the data, PLR match was defined as any given observation wherein NPi ≥ 3 and CV ≥ 0.8 mm/s or NPi < 3 and CV < 0.8 mm/s. Mismatch therefore indicates that one measured (NPi or CV) value is in the opposite direction (e.g., NPi ≥ 3 and CV < 0.8 mm/s). A congruency table chi-squared statistics was carried out to assess the match-mismatch dynamics between a reading that connotes a normal NPi output versus a normal CV and vice versa. A Cochran–Mantel–Haenszel test and a Fisher’s exact test was added on to the analyses to lend reliability to the significance of the stratified binary variables. The categorical variable was re-categorized into four groups and juxtaposed to other variables in the END PANIC registry like size of pupils, age, and gender, in order to explore observations of normal NPi and sluggish pupils, or abnormal NPi and brisk pupils. Next, a glimmixed regression of the original dichotomous categorical variables of NPi versus CV was plotted to further elucidate the covariance and relationship between the two variables. A repeated measure and size control was enacted by applying these variables to the glimixed model: NPi = CV. Finally, the data was re-dichotomized and computed in another glimmixed regression that reveals the odds ratio of getting an abnormal reading of CV when there is a normal reading for NPi and vice versa.

### Trial Registration

The trial is registered with clinical trials NCT02804438.

## Results

There were 27,462 readings from 1,617 subjects; there was a mean of 17.0 (SD = 41) readings per subject. Of these subjects were diagnosed as acute ischemic stroke (AIS) (n = 284, 19.0%), intracaerbral hemorrhage (ICH) (n = 183, 12.2%), subarachnoid hemorrhage (SAH) (n = 128, 8.6%), tumor (n = 385, 25.8%), and other neurological or neurosurgical diagnosis (NSU) (n = 515, 34.5%). Subject mean age was 57.7 (SD = 17.2) years with 808 (49.97%) males. Subjects self-identified race as either Caucasian (n = 1157, 71.6%), African American (n = 270, 16.7%), Asian (n = 50, 3.1%), Pacific Islanders (n = 83, 5.1%) or other (n = 84, 53.2) (56, 3.5% failed to report a race). Of the readings, 13,691 readings (50.2%) were from females and 13,771 observations (49.9%) were from males.

### Neurologic pupil index

There was a statistically significant but not clinically relevant difference in mean NPi for the left vs right eye [μ = 4.10, SD = 0.98 (CI: 4.0–4.19) vs μ = 4.15, SD = 0.90 (CI: 4.07–4.23) respectively α = 0.05 p < 0.0001]. There was a statistically significant, but not clinically relevant difference in right eye mean NPi values comparing females (μ = 3.8, SD = 1.2) and males (μ = 4.0, SD = 1.2; p < 0.0001). This was similar comparing left eye mean Npi values for males (μ = 3.9, SD = 1.3) and females (μ = 3.7, SD = 1.3; p < 0.001). Age accounted for 5.0% of the variance in right eye Npi values (r^2^ = 0.0477, p < 0.0001) and 8.0% of the variance in left eye Npi values (r^2^ = 0.0795 p < 0.0001). There was a statistically significant difference in an omnibus test of left eye mean Npi by diagnosis (AIS μ = 4.0, ICH μ = 3.9, tumor μ = 3.7, SAH μ = 3.7, NSU μ = 3.8; p < 0.0001); and also for right eye mean Npi by diagnosis (AIS μ = 4.1, ICH μ = 3.9, tumor μ = 3.8, SAH μ = 4.0, NSU μ = 3.9; p < 0.001).

### Constriction velocity

There was a statistically significant, but not clinically relevant, difference in mean CV comparing the left eye (μ = 1.5 mm/s, SD = 0.9, 95% CI: 1.52–1.54) and right eye (μ = 1.5 mm/s, SD = 0.9, 95% CI: 1.50 −0 1.52; p = 0.0194). There was a statistically significant difference in right eye mean CV comparing females (μ = 1.5 mm/s, SD = 0.92) and males (μ = 1.48 mm/s, SD = 0.82; p < 0.0001); and in left eye mean CV comparing females (μ = 1.6 mm/s, SD = 0.91) and males (μ = 1.5 mm/s, SD = 0.82; p < 0.0001). Age accounted for <1%% of the variance in right eye CV (r^2^ = 0.0039, p < 0.0001), and <1% of the variance in left eye CV (r^2^ = 0.0013, p < 0.0001). There was a statistically significant, but not clinically relevant, difference in an omnibus test of left eye mean CV by diagnosis (AIS [μ = 1.7], ICH [μ = 1.5], SAH [μ = 1.5], tumor [μ = 1.5], SH [μ = 1.5], and NSU [μ = 1.5]; p < 0.0001); and right eye mean CV by diagnosis (AIS [μ = 1.6], ICH [μ = 1.4], tumor [μ = 1.6], SH [μ = 1.5], and NSU [μ = 1.5]; p < 0.001).

### NPi and CV associations

Of 27,462 readings, 13,527 (49.2%) readings from 221 subjects were classified as having both brisk CV (≥0.8 mm/s) and normal NPi (≥3.0) bilaterally (Table 1). Similarly, there were 2,954 (10.8%) observations from 707 subjects with bilaterally abnormal NPi and slow CV. There were 2,494 (9.1%) in which one pupil was found to have a normal NPi and brisk CV where the opposite pupil had an abnormal NPi and slow CV. For the purpose of this study, a mismatch was defined as any observance of a normal NPi (≥3.0) and a slow CV (<0.8 mm/s) or abnormal NPi (<3.0) and brisk CV (≥0.8 mm/s). There were 8,487 (30.9%) observations categorized as mismatch. The most common mismatch (normal NPi and slow CV), was noted in 2,301 (8.4%) of all observations in 179 subjects. Moreover, 659 (40.8%) of patients had at least one observation categorized as a mismatch. None of the 2,197 observations of pupils with NPi = 0 had any CV (CV = none), indicating a non-reactive pupil; the reverse was also true in that there were no observations of CV = 0 where NPi was >0.

**Table 1:** Paired observations for left and right eye NPi and CV readings.

			Left Eye				*Row total*
			NPi ≥ 3		NPi < 3		
			CV ≥ 0.8	CV < 0.8	CV ≥ 0.8	CV < 0.8	
Right Eye	NPi ≥ 3	CV ≥ 0.8	13,527 (221)	1,184 (107)	600 (44)	1,481 (17)	16,792
		CV < 0.8	1,223 (117)	2,301 (179)	56 (5)	895 (51)	4,475
	NPi < 3	CV ≥ 0.8	503 (42)	28 (2)	512 (25)	377 (25)	1,420
		CV < 0.8	1,013 (13)	522 (33)	286 (29)	2,954 (707)	4,775
Column *total*			16,266	4,035	1,454	5,707	27,462

*Values represent observations and (subjects) with at least one observation).

Table 2 presents data on PLR reading match (NPi ≥ 3 and CV ≥ 0.8 mm/s or NPi < 3 and CV < 0.8 mm/s) for each individual paired Glasgow Coma Score (GCS) category (best verbal response, best eye response, and best motor response). As expected the most common observations for PLR match were noted for higher GCS scores at both on admission and at discharge (p < 0.001).

**Table 2:** Distribution of Matched* observations by Glasgow Coma Scores on admission and at discharge.

Glasgow Coma Score		On Admission Match		At Discharge Match	
		No	Yes	No	Yes
Best Verbal Response Score	1	5,603 (38.0%)	9152 (62.0%)	3235 (40.7%)	4708 (59.3%)
	2	252 (28.6%)	630 (69.8%)	985 (37.1%)	1671 (62.9%)
	3	137 (20.4%)	534 (79.6%)	1175 (26.0%)	3340 (74.0%)
	4	719 (24.9%)	2174 (75.2%)	870 (16.2%)	4494 (83.8%)
	5	1751 (21.4%)	6444 (78.6%)	28 (13.7%)	177 (86.3%)
Best Eye Opening score	1	3768 (41.6%)	5290 (58.4%)	5431 (34.9%)	10143 (65.1%)
	2	1191 (41.9%)	1649 (58.1%)	148 (27.7%)	387 (72.3%)
	3	911 (21.4%)	3,349 (78.6%)	90 (22.7%)	301 (77.0%)
	4	1,879 (23.1%)	8658 (76.9%)	315 (21.6%)	1142 (78.4%)
Best Motor Response Score	1	1718 (39.6%)	2619 (60.4%)	1511 (39.5%)	2311 (60.5%)
	2	586 (45.3%)	707 (54.7%)	296 (46.3%)	343 (53.7%)
	3	394 (35.7%)	710 (64.3%)	234 (37.6%)	389 (62.4%)
	4	1193 (33.1%)	2409 (66.9%)	1106 (36.8%)	1899 (63.2%)
	5	1266 (33.7%)	2492 (66.3%)	1221 (33.3%)	2451 (66.8%)
	6	3289 (24.8%)	9959 (36.3%)	1818 (20.9%)	6902 (79.2%)

^*^Match indicates that for any given observation, NPi ≥ 3 and CV ≥ 0.8 or NPi < 3 and CV < 0.8.

There were 51 subjects with at least one observation of NPi = 0 bilaterally. Of these, 33 (64.7%) died prior to discharge, 8 (15.7%) were discharged with modified Rankin Score (mRS) >2; 2 were discharged with mRS 0–2; we were unable to determine mRS for 8 subjects with functional status not documented at discharge. Observing a CV ≥ 0.8 was associated with high likelihood of observing a normal (≥3) NPi in both the left eye (OR = 12.6, 95% CI = 11.8–13.5), and right eye (OR = 9.6, 95% CI = 8.9–10.3). Observing a CV < 0.8 was associated with high likelihood of observing an abnormal (<3) NPi in both the left eye (OR = 12.6, 95% CI = 11.8–13.5)) and right eye (OR = 9.6, 95% CI = 8.9–10.3). Similarly, observing a slow CV (<0.8 mm/s) was associated with low likelihood of observing a normal NPi in both the left eye (OR = 0.08, 95% CI = 0.07–0.09), and right eye (OR = 0.1, 95% CI = 0.1–0.1). Observing a brisk CV (≥0.8 mm/s) was associated with low likelihood of observing an abnormal NPi (<3) in both the left eye (OR = 0.08, 95% CI = 0.07–0.09), and right eye (OR = 0.1, 95% CI = 0.1–0.1). Simple regression was used to explore the NPi and CV relationship further. There was a weak association between NPi and CV for both the left (r^2^ = 0.068) and right (r^2^ = 0.052) eyes (p < 0.001). In the left eye, increasing pupil size and the percent change in pupil size were predictive of CV (r^2^ = 0.72 and r^2^ = 0.457, p < 0.001; respectively); this was similar for the right eye (r^2^ = 0.725 and r^2^ = 0.379, p < 0.001; respectively).

Table 1 provides a summary of paired left and right eye observations. Not surprisingly, the most common finding (13,527 observations in 221 subjects) was a bilaterally normal PLR (NPi > 3.0 and reactive CV > 0.8 mm/s); followed by the finding (2,954 observations in 707 subjects) of bilaterally abnormal PLR (NPi < 3 and CV < 0.8 mm/s). The table also provides evidence that unilateral findings, including CV-NPi mismatch, are relatively common, and comprise 30.9% (8,487/27,462) of observations in this sample. Similarly, CV-NPi mismatch is more common with lower GCS scores (Table 2). However, in our sample, there was no association between admission diagnosis and frequency of CV-NPi mismatch.

## Discussion

The finding of high numbers of observed mismatch (e.g. NPi < 3.0 and CV > −0.8 mm/s) call into question long held beliefs that a brisk pupil is sufficient evidence to rule out abnormal PLR. Regression modeling extends findings from Ellis^22^ that CV is a nonlinear function partially dependent on initial pupil size and the percent change in pupil size in response to light stimuli. Clinical observations support this paradigm: a pupil constricting from 5 mm to 2 mm over 1 second (3 mm change in 1 sec) will have a higher CV than a pupil constricting from 2 mm to 1.5 mm over the same period of time (0.5 mm change in 1 sec). Yet, both may reflect a normal PLR.

Human observations have limitations^6^. Observation of a change in size from 2 mm to 1.5 mm is difficult for most human observers, increasing the likelihood that this pupil would be scored sluggish or potentially fixed despite a normal NPi. An example supporting NPI < 3 with CV > 0.8 mm/s is the patient with 9 mm pupil that constricts briskly to 8 mm. Few would argue that a change from 9 to 8 mm is normal, yet the CV would be >0.8 mm/s and likely be easily observed by the human eye. Hence, a sluggish pupil can be observed in a patient who is absent of cranial nerve injury, and a brisk pupil does not rule out cranial nerve injury. For example, a patient with a cranial nerve injury could present with a brisk CV, but an abnormal PLR. If the brisk CV is misconstrued as a normal PLR, the clinician delays to investigate the patient’s cranial nerve function further, whereas, earlier intervention could be vital to a better prognosis.

The tradition of relying upon constriction speed (brisk, sluggish, fixed), does not adequately account for a diverse number of variables involved in the PLR. The CV, while approximating subjective scoring of constriction speed, is inadequate to serve as a sole measure upon which to validate functional status of cranial nerves II and III. However, because CV is determined in part due to benign variables such as ambient light and baseline pupil size, there is no agreed upon definition of normal nor abnormal CV value. In this study, we opted to dichotomize CV at 0.8 mm/s based on preclinical, historical evidence and clinical experience as roughly 2 standard deviations from mean values in healthy populations^23,26–28^. The use of 0.8 mm/sec as a cutpoint is therefore suspect and future research is needed to more adequately interpret findings.

These data support the need for in-depth analysis with significantly larger cohorts and greater temporal resolution of PLR findings and clinical correlates. Differences in observed independent mean NPi and CV values for males and females is likely due to large sample sizes, and a proportion of the variance in values is explained by pupil size prior to PLR.

Likewise, it is not surprising that a pupil can have a brisk reaction (fast CV) but still not be considered a normal PLR. The pathophysiological explanation here makes sense because CV and NPi evaluate two different aspects of the PLR. The NPi evaluates a host of variables (Latency, CV, mean CV, size at baseline, percent change, and dilation velocity) and compares (indexes) that value to a standard set of normal values. Whereas, the CV only accounts for the displacement of the pupils with respect to time. This is most easily observed in the patient with large pupils.

Lower CV given that the NPi is normal appears to be the most prevalent in Caucasian males with dark eye colors, who have low initial GCS scores. This indicates that the neurodynamic assessment of CV and NPi might follow different components of the brain. Moreover, the results support that the CV-NPi relationship has the potential to be an intriguing non-invasive biomarker which may open up a host of other potential biomarkers like latency, percent change in size, and dilation velocity.

### Limitations

There is a possible confounding influence that is based on the repeated measures. This confounding factor was addressed by carrying out a regression procedure that controlled for repeated measures, which were classified by a variable called time point. At the entry of the data, a time point variable was collected to indicate that the time that the data was collected. This time point variable was used as a class to control a regression of NPi and CV. For the left eye, there was a log likelihood ratio of 39,436.10 (p < 0.0001) for NPi = CV. After controlling for size, the likelihood ratio decreased to a value of 31,407.62 (p < 0.0001). Likewise, for the right eye, there was a log likelihood of 42,507.90 (p < 0.0001), and after controlling for size, the likelihood ratio decreased to a value of 32,789.66 (p < 0.0001).

Different pathologies may be more or less susceptible to PLR changes. A large infratentorial hemorrhage, for instance, would be more likely to impact PLR than a small ischemic parietal lobe stroke. Although, in our sample, admission diagnosis was not associated with increased likelihood of CV-NPi mismatch, this sample represents a heterogeneous sample and future research is important to confirm and extend these results for specific pathologies. There is an implicit margin of error pertaining to the devices that were used to collect pupil readings. Variations in battery level, lamp and ambient light could have been standardized across all subjects. Also, there were no recorded variables to control for optic nerve damage, which is a potential confounder considering that the subjects of this study had surgeries that could have contributed to optic nerve damage.

## Conclusion

The finding of a briskly reactive pupil is insufficient to conclude that the PLR is normal. While 83% of readings in this sample indicated that interpreting CV as brisk, or sluggish would match the interpretation for NPi as normal or abnormal, the 17% mismatch bears a clinical significance. Practitioners should consider the implications of these findings whenever they interpret readings from the automated pupilometry. Additional research is required to determine the utility of CV-NPi as a potential biomarker.
